# Case Report of HIV and Neurosyphilis Coinfection in a Recent Migrant: Old Diseases in New Faces

**DOI:** 10.5811/cpcem.20943

**Published:** 2024-11-25

**Authors:** Alexa Van Besien, Ian Paolo Mauricio, Ronald Belfort, Joseph Lykins

**Affiliations:** * Chobanian & Avedisian School of Medicine, Department of Emergency Medicine, Boston University, Boston, Massachusetts; † Boston Medical Center, Interpretive Services, Boston, Massachusetts

**Keywords:** neurosyphilis, Treponema pallidum, HIV, coinfection, Haiti, case report

## Abstract

**Introduction:**

Coinfection with human immunodeficiency virus (HIV) and *Treponema pallidum* represents a unique challenge in management, with increased risk of neurological complications. Haiti is well-known for being disproportionately impacted by the HIV epidemic, with rates of infection ~6 times higher than in the United States (US). Rates of coinfection in Haiti are incompletely characterized but likely high. The US has seen a marked increase in migration from Haiti, with implications for public health and migrant health management.

**Case Report:**

A 69-year-old male, recent Haitian migrant presented for subacute altered mental status and visual and auditory hallucinations for approximately four weeks. The patient’s neurological exam was non-focal, but laboratory evaluation showed an elevated paraprotein gap (6.7 grams per deciliter). This prompted concern for infectious etiology. The patient was diagnosed with HIV/AIDS with a CD4+ count of 154 cells per cubic millimeter and a positive rapid plasma reagin test (titer 1:128), with cerebrospinal fluid demonstrating elevated white blood cell count and protein concentration, consistent with neurosyphilis. The patient completed 14 days of intravenous benzathine penicillin G, with hospitalization complicated by hyponatremia and vomiting, which resolved after antibiotics.

**Conclusion:**

This case highlights the risk of coinfection with HIV and neurosyphilis in the Haitian migrant population and suggests possible benefit in routine screening for HIV and syphilis in the emergency department, particularly for at-risk populations. Neurosyphilis can be difficult to diagnose, requiring a high index of suspicion. Migrant patients can have difficulty accessing healthcare services, and the emergency department may have a role in screening and initiation of treatment in this population.

## INTRODUCTION

*Treponema pallidum*, the spirochete causing syphilis, can invade the central nervous system (CNS), affecting brain, eyes, spinal cord, and meninges. Presence of *T pallidum* in cerebrospinal fluid can occur at any stage of infection, whether symptomatic or not. Neurosyphilis within the first year of infection typically presents with meningitis, but it has a more varied presentation as the organism persists in the CNS, including meningovascular and parenchymatous lesions, CNS gummata, and ocular syphilis.^1^ The presentation of neurosyphilis is often non-specific and can mimic microvascular and neurodegenerative disorders, infectious encephalitides, complications of substance use, and primary HIV-induced neurocognitive decline. These conditions are frequently seen in geriatric patients and potentially present with neuropsychiatric symptoms.

Rates of primary and secondary syphilis have risen steadily, and the United States Centers for Disease Control and Prevention (CDC) estimates that from 2020 to 2021 rates of syphilis cases in the United States (US) increased 32%.^2^ Neurosyphilis is particularly prevalent among HIV-positive individuals with untreated syphilis, as high as 40%.^3^ The burden of this syndemic is distributed unequally across the globe, with Haiti being disproportionately impacted. Many Haitians face severe stigma, limiting access to already limited care.^4^ Certain populations within Haiti are particularly affected, including sex workers and sexual and gender minorities.^5^ Data is sparse on rates of syphilis in Haiti. Increased Haitian immigration to the US makes these questions relevant to US clinicians, particularly those in the emergency department (ED), where many recently arrived migrants experience their first contact with the US healthcare system.^6^

Our case (see Figure for hospital course) highlights the need for a high index of suspicion for coinfection of HIV and syphilis, particularly in geriatric patients and recently arrived migrants who might be at elevated risk and whose healthcare access can be limited. It impresses a call to action for emergency physicians to take a more public health approach to our work, addressing challenges through novel approaches such as routine disease screening from the ED for vulnerable populations.

## CASE REPORT

A 69-year-old Haitian immigrant male with hypertension presented to the ED with his daughter for altered mental status, neck pain, and hallucinations. He had not seen a physician for over 20 years. The patient was alert and oriented to person and place but not to time. He complained of three days of paraspinal neck pain but denied recent trauma, photophobia, new incontinence, back pain, skin changes, or focal neurological changes. The patient’s daughter reported a four-week decline in his mental status, including increased confusion, disorientation to time, and visual and auditory hallucinations of deceased family members.

His ED vital signs were heart rate 61 beats per minute, blood pressure 200/94 millimeters of mercury, respiratory rate 17 breaths per minute, oxygen saturation 98% on room air, and temperature 98.4° Fahrenheit. He had full extraocular motility without anisocoria, afferent pupillary defect, or Argyll Robertson pupils. The patient’s neck was supple without meningismus. Cranial nerves were intact, strength was 5/5, and sensation was intact in the bilateral upper and lower extremities. Cerebellar testing and gait were normal. Given the patient’s non-specific presentation with recent arrival to the US four weeks prior, a broad diagnostic evaluation was initiated.

Initial labs showed mild normocytic anemia (hemoglobin 12.8 grams per deciliter [g/dL]) (13.5–17.5 g/dL) without leukocytosis. Comprehensive metabolic panel was unremarkable with the exception of mild hyponatremia (132 millimoles per liter (mmol/L), mild hyperglycemia (133 milligrams per deciliter [mg/dL]), and elevated (normal < 4 g/dL) serum protein (10.3 g/dL) (6.8–8.6 g/dL). With a normal serum albumin (3.6 g/dL) (3.5–5.0 g/dL), the patient had a calculated paraprotein gap of 6.7 g/dL, which was elevated. Thyroid stimulating hormone, and vitamin B_12_ levels were normal. Serum toxicology screen for tricyclic antidepressants, salicylate, ethanol, and acetaminophen was negative. Hepatitis C antibody testing was negative. Computed tomography angiogram of the head and neck showed no acute abnormalities. Hypertensive encephalopathy was also included within the differential diagnosis but was deemed less likely in the absence of any other signs of end-organ injury. Consent was obtained for HIV and syphilis testing. Results of the rapid plasma reagin were positive at 1:128 titer, and HIV-1 antibody testing was positive. The absolute CD4^+^ count was measured at 154 cells per cubic millimeter, and the viral load was 184,000 copies per milliliter. Lumbar puncture was unsuccessful in the ED.

**CPC-EM Capsule**

What do we already know about this clinical entity?

*Coinfection between neurosyphilis and HIV can have profound impact on health, presents heterogeneously, and diagnosis is often delayed*.

What makes this presentation of disease reportable?

*This case presents in a recent migrant to the US from Haiti, likely with significantly delayed diagnosis and possible long-term neurocognitive implications*.

What is the major learning point?

*Recently arrived migrants experience barriers to care. Neurosyphilis and HIV, which can present with altered mental status, can be screened for in the ED*.

How might this improve emergency medicine practice?

*In expanding emergency physicians’ role to include bridging care for migrants, we promote health equity and diagnose conditions early, preventing long-term sequelae*.

During the patient’s hospitalization, both infectious diseases and neurology were consulted. Initial treatment for neurosyphilis was delayed secondary to difficulty obtaining cerebrospinal fluid (CSF). The patient reported possible sexual exposures but denied history of intravenous (IV) drug use or sexual contact with men. He was started on trimethoprim-sulfamethoxazole (TMP-SMX) for *Pneumocystis jirovecii* pneumonia (PJP) prophylaxis. Antiretroviral therapy (ART) was deferred initially due to risk of immune reconstitution inflammatory syndrome. The CSF was ultimately obtained by interventional radiology, revealing elevated white blood cell count (115 per microliter (μL) with polymorphonuclear predominance, an elevated total protein (85 mg/dL), and a normal glucose (45 mg/dL). Venereal Disease Research Laboratory testing of the CSF was unavailable due to lab transporting error. *Mycobacterium tuberculosis* polymerase chain reaction of CSF was negative, as was the bioMérieux BioFire meningitis/encephalitis CSF panel.

Magnetic resonance imaging (MRI) of the brain demonstrated global volume loss. The patient was prescribed vitamin B_12_ and a 14-day course of IV penicillin for treatment of neurosyphilis. At this point ART was again deferred due to vomiting and worsening hyponatremia (128–131 mmol/L). Nephrology was consulted, attributing the hyponatremia to medication-induced syndrome of inappropriate antidiuretic hormone from TMP-SMX. The PJP prophylaxis was transitioned to atovaquone with improvement in serum sodium. The patient was subsequently discharged on appropriate ART and prophylaxis, having completed neurosyphilis treatment. On follow-up two months after hospitalization, the patient’s daughter reported he was functioning well at home, eating and ambulating independently, but he continued to have intermittent confusion and memory loss.

## DISCUSSION

Altered mental status always necessitates a broad differential diagnosis. In this patient, it included neurosyphilis, syphilitic gummata, HIV-induced encephalopathy, herpes simplex virus encephalitis, a primary neurocognitive disorder like dementia, neoplasms, cryptococcal meningitis, and progressive multifocal leukoencephalopathy among other processes. Suspicion for HIV infection was increased due to elevated paraprotein gap noted on initial labs, as well as the prevalence of HIV in the patient’s country of origin. The paraprotein gap is calculated by the difference between total protein and serum albumin. These values are available on a hepatic panel or comprehensive metabolic panel, which are regularly ordered ED tests. A difference >4 g/dL, an elevated paraprotein gap, suggests multiple possible causes, including an underlying chronic infection such as HIV or hepatitis C virus.^7^

Syphilis is known classically as “the great imitator” with William Osler credited as saying, “He who knows syphilis knows medicine.” The patient’s subacute, rather than gradual, onset of neuropsychiatric symptoms prompted consideration of an infectious process like neurosyphilis. It should be noted that definitive diagnosis of neurosyphilis could not be made with CSF testing, although review of the literature suggests that no single test can definitively diagnose this infection in all patients. In the migrant population, this is particularly salient. One study estimated rates of syphilis among refugees to the US to be 373 per 100,000, though this may be an underestimate.^8^ Syphilis might not have been considered, however, given the patient’s age. This case provides an opportunity to discuss the “desexualization” of geriatric patients, making assumptions about their sexual behavior and neglecting the sexual history.^9^ In a sample of older US adults, only 17% discussed sexual issues with their doctors, with 61% of these discussions being patient-initiated.^10^

Previous studies note higher syphilis prevalence among refugees and migrants in certain South American communities. This is perhaps unsurprising given limited access to sexually transmitted infection (STI) screening, comprehensive sex education, and contraceptive practices, as well as the risk of sexual violence.^11^,^12^ Furthermore, it highlights how migration contributes to acquisition of HIV and other STIs, with increased proportions of infections in high-income countries stemming, at least in part, from migrants from lower or middle-income countries.^13^ This case emphasizes the vigilance that emergency physicians must bring to care of migrant and refugee patients, with high indexes of suspicion for STIs especially for individuals who have barriers to access. It highlights the need for increased sexual health services for migrants, as well as commitment at physician, community, and policy levels to provide culturally appropriate prevention and healthcare to these often-marginalized communities.

Routinely implemented screening for STIs and other conditions in the ED could contribute to public health, particularly for migrant patients. The CDC has recommended ED-based opt-out HIV screening since 2006, with academic medical centers leading implementation.^14^ The American College of Emergency Physicians has issued a policy statement encouraging universal opt-out HIV screening.^15^ Academic EDs comprise 3% of total EDs but account for the majority of sites providing HIV screening, with roughly 1 in 4 providing targeted screening and almost 1 in 5 providing nontargeted screening in a national survey performed in 2009.^16^,^17^ Benefits of screening include identifying patients with acute HIV at higher risk for viral transmission, indicating added public health benefit.^18^ This study included sites in Illinois, Texas, California, Louisiana, and Pennsylvania, indicating the practice has spread across the US.

Screening for syphilis in the ED, a more novel proposition, is a natural outgrowth of HIV testing. Previous data suggests syphilis is often ignored at visits testing for gonorrhea and *Chlamydia*.^19^ One study found routine syphilis screening feasible and noted high rates of infection even in groups typically considered “low risk.”^20^ Implementing and improving screening programs in community EDs will be vital for equitable care, meeting patients where they are and addressing their medical needs when other parts of the healthcare system cannot or will not.

Notably, our patient presented with hallucinations. Our literature review found that <20% of neurosyphilis cases present with psychiatric symptoms, including paranoia, behavioral changes, hallucinations, mania, and cognitive impairment.^21^ A case series revealed that multiple patients were admitted to psychiatric units before diagnosis of neurosyphilis was established.^22^ This illustrates that, especially in older patients without pre-existing psychiatric history, new-onset neuropsychiatric symptoms require a broader differential, and in the appropriate clinical context CSF examination must be considered.^23^ This is especially true for recent migrants from high-risk areas and patients with known comorbid HIV infection.

This case highlights the impact of shared population experience on ED clinical decision-making. The patient’s status as a recent migrant from a high-prevalence area with a comparatively fragmented healthcare system prompted consideration of HIV and neurosyphilis as potential explanations for his subacute presentation. Best practices from this case include early involvement of interpreter services to interpret not simply the words being communicated but the cultural context in which such communication occurs. Our own interpreter team suggests the importance of coordination with interpretive services prior to meeting the patient, a “pre-brief” in which goals of the encounter, clinician concerns about cognition, and other factors might be communicated, such that the interpreter can assess for culturally relevant factors and medically relevant details.

## CONCLUSION

We add to the literature a case of neurosyphilis—novel in highlighting social determinants of health for immigrant populations with high HIV prevalence. In keeping with its status as “the great imitator,” syphilis should remain high on the differential, particularly in undifferentiated altered mental status and new-onset neuropsychiatric abnormalities in individuals from higher prevalence areas or with risk factors. High index of suspicion for syphilis and HIV coinfection must be maintained, particularly in EDs that serve migrant populations and are often the first point of contact with the US healthcare system. This case further highlights the importance of taking a more focused public health lens to care in the ED, potentially leading to the conclusion that screening for HIV and syphilis, as is part of routine care in multiple EDs across the country, might better be viewed as standard of care, and would identify patients before they develop irreversible complications, reducing consequent morbidity and mortality.

## Figures and Tables

**Figure:**
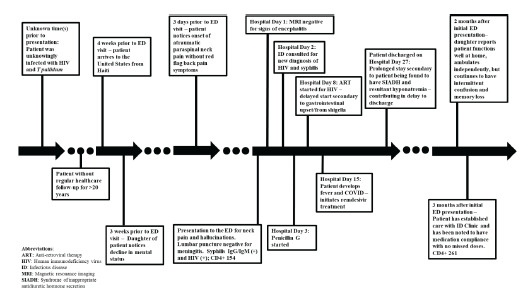
Hospital course of 6*9-*year-old Haitian immigrant coinfected with HIV and neurosyphilis. *ART*, anti-retroviral therapy; *ED*, emergency department; *ID*, infectious diseases; *SIADH*, syndrome of inappropriate antidiuretic hormone.
